# HSV Infection Induces Production of ROS, which Potentiate Signaling from Pattern Recognition Receptors: Role for S-glutathionylation of TRAF3 and 6

**DOI:** 10.1371/journal.ppat.1002250

**Published:** 2011-09-15

**Authors:** Regina Gonzalez-Dosal, Kristy A. Horan, Stine H. Rahbek, Hidenori Ichijo, Zhijian J. Chen, John J. Mieyal, Rune Hartmann, Søren R. Paludan

**Affiliations:** 1 Department of Biomedicine, Aarhus University, Aarhus, Denmark; 2 Center of Excellence Program, Japan Science and Technology Corporation, The University of Tokyo, Tokyo, Japan; 3 Department of Molecular Biology, University of Texas Southwestern Medical Center, Dallas, Texas, United States of America; 4 Department of Pharmacology, Case Western Reserve University, School of Medicine, Cleveland, Ohio, United States of America; 5 The Louis B. Stokes Veterans Affairs Medical Research Center, Cleveland, Ohio, United States of America; 6 Department of Molecular Biology, Aarhus University, Aarhus, Denmark; University of Alabama at Birmingham, United States of America

## Abstract

The innate immune response constitutes the first line of defense against infections. Pattern recognition receptors recognize pathogen structures and trigger intracellular signaling pathways leading to cytokine and chemokine expression. Reactive oxygen species (ROS) are emerging as an important regulator of some of these pathways. ROS directly interact with signaling components or induce other post-translational modifications such as S-glutathionylation, thereby altering target function. Applying live microscopy, we have demonstrated that herpes simplex virus (HSV) infection induces early production of ROS that are required for the activation of NF-κB and IRF-3 pathways and the production of type I IFNs and ISGs. All the known receptors involved in the recognition of HSV were shown to be dependent on the cellular redox levels for successful signaling. In addition, we provide biochemical evidence suggesting S-glutathionylation of TRAF family proteins to be important. In particular, by performing mutational studies we show that S-glutathionylation of a conserved cysteine residue of TRAF3 and TRAF6 is important for ROS-dependent activation of innate immune pathways. In conclusion, these findings demonstrate that ROS are essential for effective activation of signaling pathways leading to a successful innate immune response against HSV infection.

## Author Summary

Herpes simplex virus (HSV) type 1 and 2 are important human pathogens, which can give rise to severe diseases during both primary and recurrent infections. In addition to activating “classical” innate and adaptive immune responses, many infections stimulate other cellular activities such as and production of reactive oxygen species (ROS). However, there is little knowledge on the cross-talk between ROS and the innate antiviral response. In this article we show that HSV infection leads to production of ROS, and that ROS play a critical role in activation of innate immune responses to these viruses. At the mechanistic level, we show that ROS stimulate glutathionylation (a protein modification) of the signaling molecules TRAF3 and 6, which promotes redox-sensitive signaling. Our data support the idea that the innate immune system not only detects specific HSV molecules but also senses the cellular oxidative stress level, and integrates this into the innate immune response to infections.

## Introduction

The innate immune response constitutes the first line of defense against invading pathogens, and relies on pattern recognition receptors (PRR)s for detection of infections through recognition of either molecular structures specific for non-self, or aberrant localization of molecules used by both host and microbe [1], [2]. Toll-like receptors (TLR)s are membrane-bound PRRs localized in the plasma membrane and endosomes, which recognize microbes at these sites. Other families of PRRs are localized in the cytoplasm, such as Retinoic acid-inducible gene (RIG)-I-like receptors (RLR)s which detect cytosolic RNA [1], [2]. The RLRs, including RIG-I and Melanoma differentiation-associated gene-5 (Mda-5), signal from the outer mitochondrial membrane via anchoring of the mitochondrial RLR adaptor molecule, MAVS (mitochondrial antiviral signaling protein) [3]. Cytosolic DNA potently stimulates innate immunity through a series of DNA receptors (DNAR)s including the AIM2-like receptors (ALR)s [3]–[7].

Herpes simplex virus (HSV) is a significant human pathogen, and whilst HSV-1 is an important cause of viral encephalitis, HSV-2 predominantly causes genital infections [8]. HSV-1 and 2 are closely related at the genetic level and accordingly share many biological and pathological properties [8]. HSV is recognized by TLR2 and TLR9 [9], [10], which act synergistically to control HSV infection in the brain in mice [11]. In humans, a dominant-negative TLR3 allele has been reported in otherwise healthy children with HSV-1 encephalitis [12]. In addition to TLRs, both RLRs and DNARs have been implicated in recognition of herpes viruses [3], [5]–[7], [13]. Most recently, we have identified γ-interferon-inducible protein 16 (IFI16) as a novel intracellular sensor of HSV DNA and mediator of expression of type I interferon (IFN) and inflammatory cytokines [7]. Thus, innate recognition of HSV involves a large spectrum of PRRs, which together orchestrate the innate immune response to infections by this virus [reviewed in ref. 14].

The innate immune system interacts closely with basic cellular processes such as autophagy and reactive oxygen species (ROS) [15], [16], thereby coordinating these cellular processes with the innate immune response. Oxygen is consumed during many cellular processes in a manner leading to production of superoxide anions (O_2_^-^) [17]–[19], which are readily converted to hydrogen peroxide (H_2_O_2_) by a reaction catalysed by superoxide dismutases. Both superoxide anions and hydrogen peroxide can be converted to other reactive species (*e.g*., hydroxyl radical, peroxynitrite, *etc.*) through reactions with a range of reactive molecules in the cell. ROS is a collective term for superoxide and the downstream-derived ions and small molecule metabolites. Due to the highly reactive nature of ROS that can modify any cellular macromolecule, the cell contains several scavengers, called antioxidants, as well as enzymes that maintain cellular redox homeostasis. Glutathione (GSH) is the most abundant cellular antioxidant. GSH can form molecular disulfides with the thiol group of the cysteine residues in oxidative conditions, thereby regulating protein function [20]. This modification has been shown to be essential for the function of several proteins such as c-jun, p50/NF-κB and IκB kinase subunit (IKK) β [21]–[23]. It has long been known that ROS play an important part in the innate immune system as a microbicidal compound produced by NADPH oxidases (NOXs) in phagosomes of phagocytic cell types such as macrophages and neutrophils [18]. More recently, it has been demonstrated that ROS are also involved in activation and regulation of a wider range of processes in the innate immune system, including autophagy, signal transduction, gene expression, activation of the inflammasome, and programmed necrosis [15], [16], [24]–[28]. Thus, cellular sensing of the intracellular redox status impacts on the immune response to infectious agents.

The recognition of viruses by the immune cells is a very complex process in which several signal transduction pathways regulating numerous cellular processes are involved. Moreover, the recent observation of the involvement of ROS as a regulator of these processes gives an additional layer of complexity that needs to be described. In this work, we demonstrate that HSV infection induces an early production of ROS, which regulates the activation of innate immune responses initiated by HSV infection, via TLRs, RLRs, and IFI16. Mechanistically, we show that ROS induces S-glutathionylation of tumor necrosis factor receptor-associated factor (TRAF) 3 and 6, which is essential for optimal stimulation of the cellular response to innate HSV recognition.

## Results

### HSV-induced ROS formation is essential for the activation of the innate immune response

**Figure 1:**
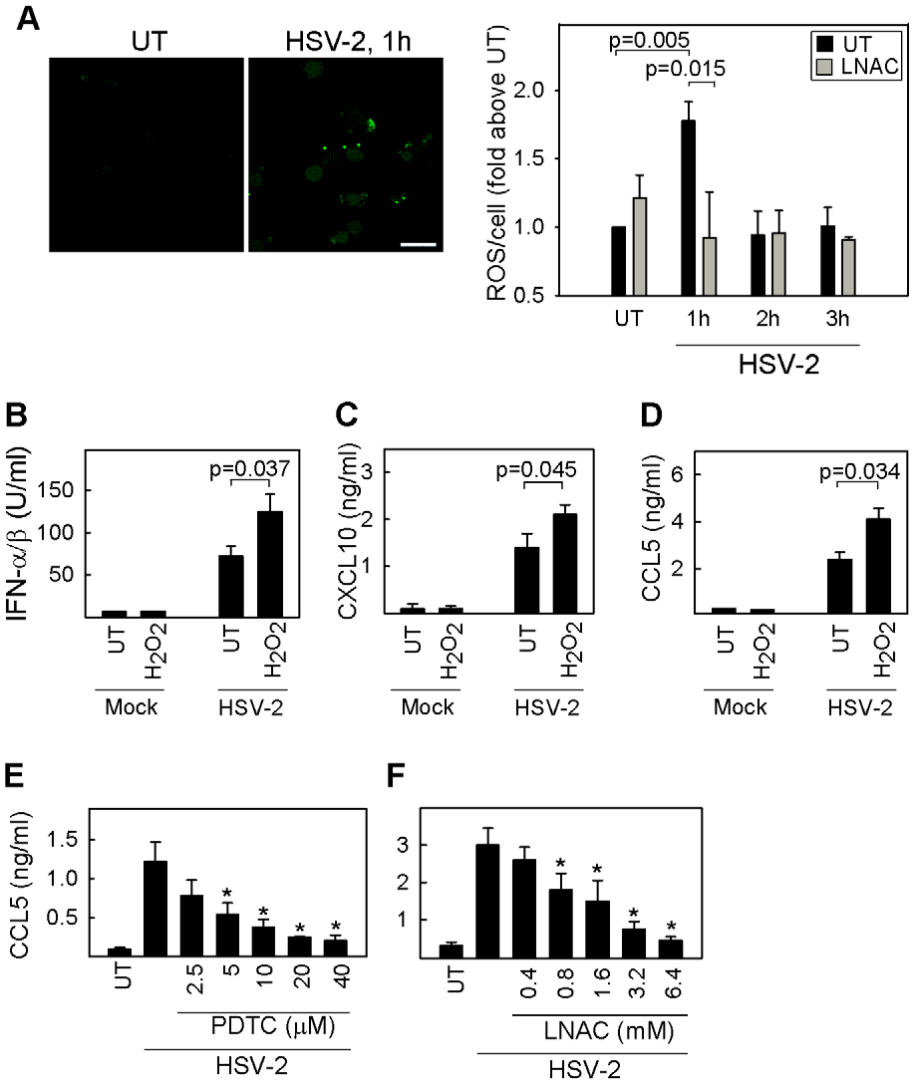
Intracellular ROS produced following HSV infection is essential for cytokine expression. (A) RAW264.7 cells were seeded in chambered-cover slides, either pre-treated with 6.4mM LNAC or complete media for 30 min, and subsequently infected with HSV-2 for 1, 2, or 3 h. CM-H_2_DCFDA (5 µM) was added immediately before imaging. ROS production was monitored in z-stack images (1 µm) and images were acquired with a 40x objective every 30 seconds by confocal microscopy. Images represent maximum intensity projections. Amount of ROS was determined and represented as mean fluorescence per cell with a minimum of 100 cells counted per condition. Data is presented as mean of 3 experiments for the untreated samples and of 2 experiments for the LNAC samples +/- sem. (B–D) Peritoneal macrophages were seeded and either left uninfected or infected with HSV-2 (3×10^6^ pfu/ml, MOI 3); each in the absence or presence of 10 µM hydrogen peroxide. Supernatants were harvested after 16 h for measurement of IFN-α/β, CXCL10, and CCL5. (E, F) Peritoneal macrophages were pre-incubated with increasing concentrations of PDTC or LNAC and subsequently infected with HSV-2 (3×10^6^ pfu/ml, MOI 3). Supernatants were harvested 12 h post infection and CCL5 protein was measured by ELISA. The data is shown as mean of 3 replicates +/- st. dev. and is representative of 3 experiments. CCL5 levels significantly different from those induced by HSV-2 alone (p<0.05) are indicated with *.

ROS has gained increasing importance in the role as a second messenger in the activation of signaling pathways. In order to examine how these radicals are involved in the recognition of HSV, we first examined if HSV infection induced formation of cellular ROS. RAW264.7 cells were infected with HSV-2 and ROS formation at different time points was monitored by live microscopy using the oxidant-sensitive fluorescent probe CM-DCFDA. ROS production was induced 1 h post-infection (Figure 1A), and this was abrogated by pre-treating the cells with the antioxidant N-acetyl-L-cysteine (LNAC), which can scavenge endogenous ROS (Figure 1A). LNAC did not affect viral entry as determined by staining for viral capsids in the cytoplasm following infection (data not shown). Next, the role of ROS in HSV-stimulated cytokine expression was examined. We first investigated the effect of exogenous ROS on expression of IFNs and IFN-stimulated genes (ISG)s in response to HSV infection. Murine primary macrophages responded to HSV-2 infection with production of type I IFN (Figure 1B) and the IFN-inducible chemokines CXCL10 (Figure 1C) and CCL5 (Figure 1D), and in all cases exogenous hydrogen peroxide (H_2_O_2_) lead to a modest but significant elevation of this response. On the contrary, pre-treatment with the general antioxidants pyrrolidine dithiocarbamate (PDTC) and LNAC strongly inhibited the cytokine response following infection with HSV-2 (Figure 1E, F). All small molecule inhibitors were used in concentrations not affecting cell viability as determined by annexin V and propidium iodide staining in macrophages (data not shown).

**Figure 2:**
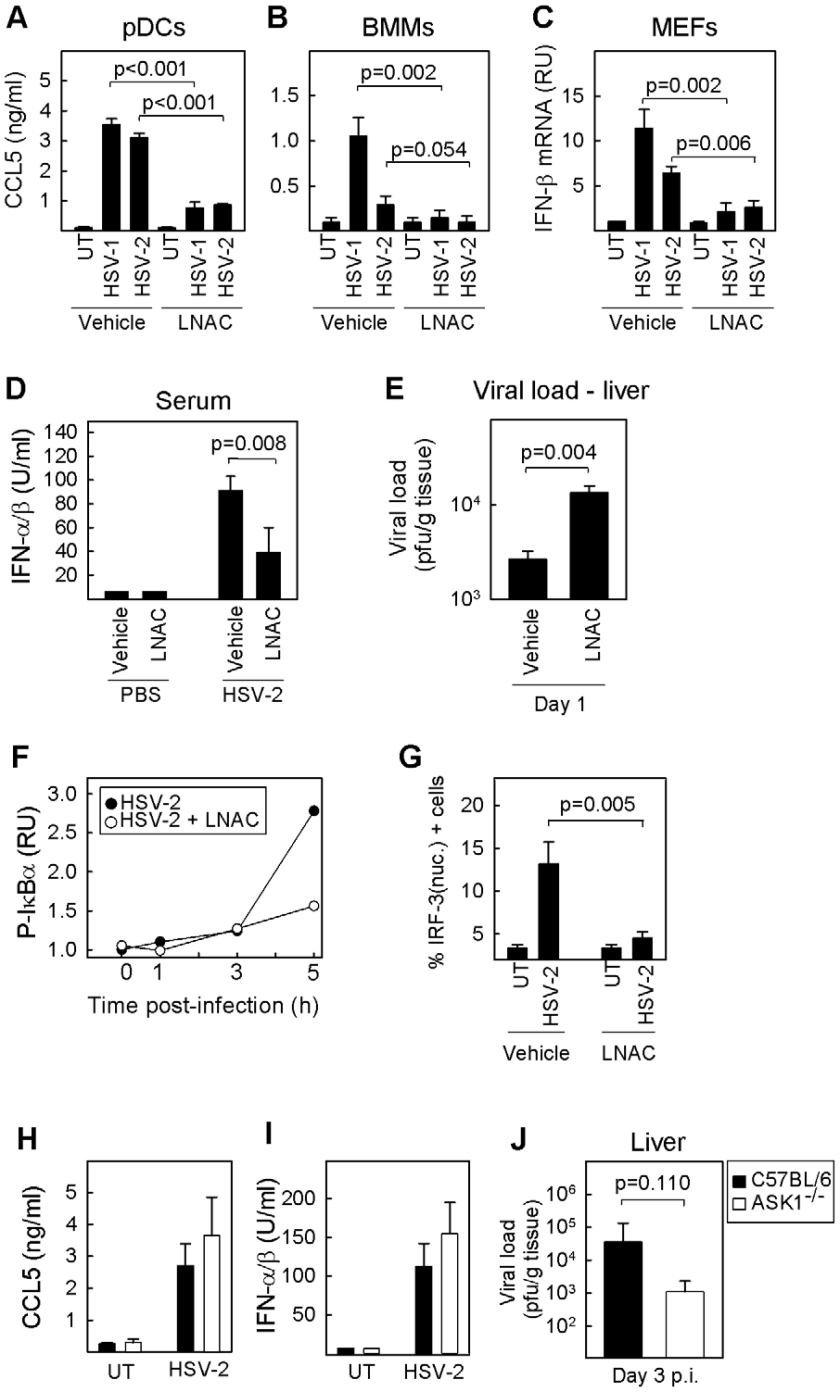
Redox-sensitive signaling following HSV recognition. (A) pDCs and (B) BMMs were treated with LNAC (3.2 mM) 30 min prior to infection with HSV-1 or 2 (MOI 3). Culture supernatants were harvested 16 h post infection, and levels of CCL5 were measured by ELISA. All data are shown as means of 3–5 replicates +/- st.dev. (C) MEFs were treated with LNAC (3.2 mM) 30 min prior to infection with HSV-1 or 2 (MOI 3). Total RNA was harvested after 6 h post-infection and IFN-β mRNA measured by real time PCR. Data is presented as means of triplicate measurements +/- st.dev. RU, relative units. (D, E) Mice were treated i.p. with LNAC (1.1 mmol/kg body weight) in saline or with saline alone followed 4 h later by infection with HSV-2 (5×10^6^ pfu). Eight and 24 hours post infection, serum and livers were harvested for measurement of type I IFN and viral load, respectively (n = 5). Data represents mean of 3–6 replicates +/- st.dev. (F, G) RAW264.7 macrophage-like cells were treated with LNAC (6.4 mM) for 30 min before infection with HSV-2 (3×10^6^ pfu/ml, MOI 3). (F) Total cell lysates were harvested at the indicated time points post infection, and phospho-IκBα was measured by Luminex. (G) The cells were fixed 3 h post infection, stained with anti-IRF-3 antibody and DAPI, and visualised by confocal microscopy. Cells were scored for nuclear IRF-3 staining. Data represent mean +/- st.dev. (H, I) Peritoneal macrophages from C57BL/6 WT and ASK1^-/-^ mice were cultured *in vitro* and infected with HSV-2 (3×10^6^ pfu/ml, MOI 3). Supernatants were harvested 16 h post infection, and the levels of CCL5 and IFN-α/β were measured. (J) C57BL/6 WT and ASK1^-/-^ mice were infected i.p. with 5×10^6^ pfu of HSV-2. Livers were isolated 3 days post infection and viral load in the organs was determined (n = 8). The data represent mean of multiple measurements +/- st.dev.

To examine how modulation of ROS levels affected the ability of HSV to activate the innate immune response in different immune cell types, we treated pDCs and macrophages with LNAC prior to infection with HSV-1 or -2. The subsequent CCL5 expression was evaluated by ELISA. Although these cell types stimulate the innate immune response against HSV infection through different combinations of PRRs, they all displayed the same inhibitory effect of the antioxidant (Figure 2A, B). Similar findings were obtained with murine embryonic fibroblasts (MEF)s treated with LNAC and infected with HSV-1 or -2 (Figure 2C).

Once the role of ROS was confirmed *in vitro*, we evaluated their potential function in innate antiviral defense *in vivo*. With this purpose, we treated mice with LNAC prior to infection with HSV-2. The virus-induced production of type I IFN was inhibited in mice receiving LNAC (Figure 2D), and these mice exhibited elevated viral load in the liver after day 1 post infection (Figure 2E). Collectively, these data demonstrate that HSV infection induces formation of cellular ROS, which are essential for the activation of an antiviral innate immune response *in vitro* and *in vivo*.

### ROS are essential for activation of NF-κB and IRF-3 signaling induced by HSV infection

The signaling pathways upstream of the transcription factors NF-κB and IRF-3 are important for establishment of innate antiviral defense against HSV [1], [2]. To clarify whether activation of these pathways was modulated by ROS, we examined how treatment with LNAC prior to HSV infection affected phosphorylation of the NF-κB inhibitor IκBα and activation of IRF-3 by nuclear translocation. We observed that the infection led to strong phosphorylation of IκBα after 5 h, and that this was potently inhibited by LNAC (Figure 2F). Likewise, HSV-induced translocation of IRF-3 to the nucleus was largely abrogated by pre-treatment with LNAC (Figure 2G). Thus, the signaling pathways activating NF-κB and IRF-3 are sensitive to the general depletion of cellular ROS.

The MAPK kinase kinase apoptosis signal-regulating kinase (ASK) 1 has been reported to be involved in ROS-dependent innate immune signaling pathways upstream of MAPKs and IRF-3 following lipopolysaccharide (LPS) treatment [16], [26]. To test the role of ASK1 in ROS-dependent innate antiviral immune responses, peritoneal macrophages from C57BL/6 and ASK1^-/-^ mice were infected with HSV-2 *in vitro*. As expected, the culture supernatants from infected cells contained elevated levels of CCL5 and IFN-α/β as compared to the supernatants from untreated cells, but no significant difference was observed between the C57BL/6 and ASK1^-/-^ cells (Figures 2H, I). Mice were infected with HSV-2 via the intraperitoneal route, and livers harvested after 3 days for analysis of viral load by plaque assay. High levels of virus were observed in the livers from C57BL/6 mice, and no impairment in the antiviral defense was observed in the ASK1 ^-/-^ mice (Figure 2J). Thus, HSV infection stimulates ROS-dependent activation of NF-κB and IRF-3 in a manner independent of ASK1.

### ROS are essential for innate antiviral immune activation via both TLRs and intracellular PRRs

**Figure 3:**
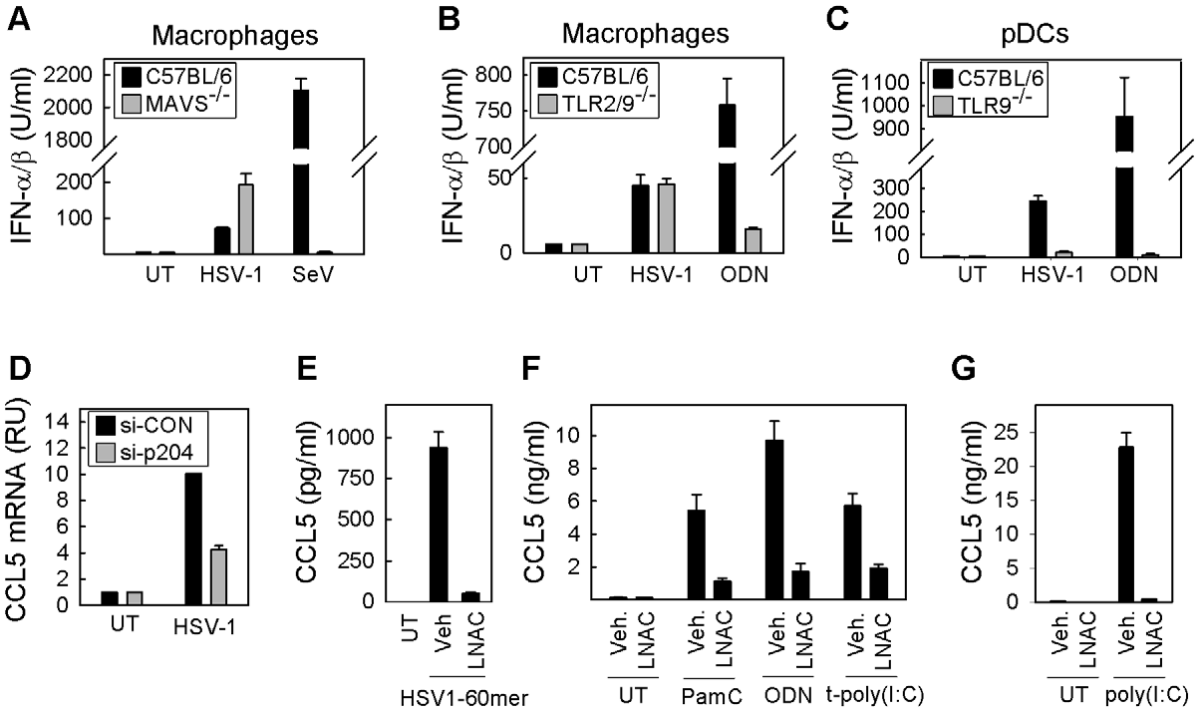
ROS and innate antiviral response: Cell-type and PRR dependence. (A, B) Macrophages were isolated from C57BL/6 WT, MAVS^-/-^ or double knock-out TLR2/9^-/-^ mice and cultured *in vitro*. Cells were treated with (A) HSV-1 KOS or Sendai Virus, or (B) HSV-1 KOS or ODN1826. Supernatants were harvested after 16 h and presence of type I IFNs were measured by bioassay. (C) pDCs were isolated from C57BL/6 WT or TLR9^-/-^ mice. Cells were treated with HSV-1 KOS or ODN1826. Supernatants were harvested after 16 h and presence of type I IFNs were measured by bioassay. (D) RAW264.7 macrophage-like cells were transfected with si-p204 or control siRNA and infected with HSV-1 KOS. Total RNA was harvested after 6 h post-infection and CCL5 mRNA measured by real time PCR. Data is presented as mean of duplicate measurements +/- st.dev. RU, relative units. (E) RAW264.7 cells were transfected with HSV1-60mer (2 µg/ml) following treatment with 6.4 mM LNAC or complete media. Culture supernatants were harvested 12 h p.i. and production of CCL5 was measured by ELISA. (F) RAW264.7 cells were seeded and treated with vehicle or LNAC (3.2 mM) 30 min prior to stimulation with Pam_3_Csk_4_ (200 ng/ml), ODN1826 (1 µM), and poly(IC):LyoVec (25 µg/ml). Culture supernatants were harvested 16 h post-stimulation, and levels of CCL5 were measured by ELISA. (G) RAW264.7 cells were incubated with poly(I∶C) (25 µg/ml) following treatment with 6.4 mM LNAC or complete media. Culture supernatants were harvested 16 h p.i. and production of CCL5 was measured by ELISA. Data represents mean of measurements from triplicate cultures +/- st.dev.

HSV stimulates the innate immune system through multiple PRRs and in cell-type specific manners [13]. In macrophages, HSV-1 induced expression of type I IFN, independently of MAVS (RLRs) (Figure 3A) and TLR2/9 (Figure 3B). We have recently shown that HSV-1 is sensed by IFI16 [7]; consistent with this, expression of CCL5 in macrophages in response to HSV-1 was dependent on the IFI16 murine ortholog, p204 (Figure 3D). In contrast, pDCs responded to the infection in a manner entirely dependent on TLR9 (Figure 3C). To further investigate the effect of ROS modulation on the activation of the PRRs that were reported to be involved in HSV recognition, we treated RAW264.7 cells with LNAC prior to stimulation with specific ligands for: cytoplasmic DNA sensors (HSV-1 60-mer), TLR2 (Pam_3_Csk_4_), TLR3 (poly(I∶C)), TLR9 (ODN1826), and RLRs (poly(I∶C):LyoVec)) (Figure 3E–G). Pre-incubation with LNAC strongly diminishes CCL5 production in response to HSV-1 60mer (Figure 3E). Similarly, stimulation via TLRs and RLRs induced a strong expression of CCL5, which was abrogated by pre-treatment with LNAC (Figure 3F, G). Collectively, these data demonstrate that all the PRRs reported to be involved in recognition of HSV are inhibited by general depletion of cellular ROS, indicating a positive role for ROS in the regulation of the innate immune response to HSV.

### ROS mediate glutathionylation of TRAF family proteins

**Figure 4:**
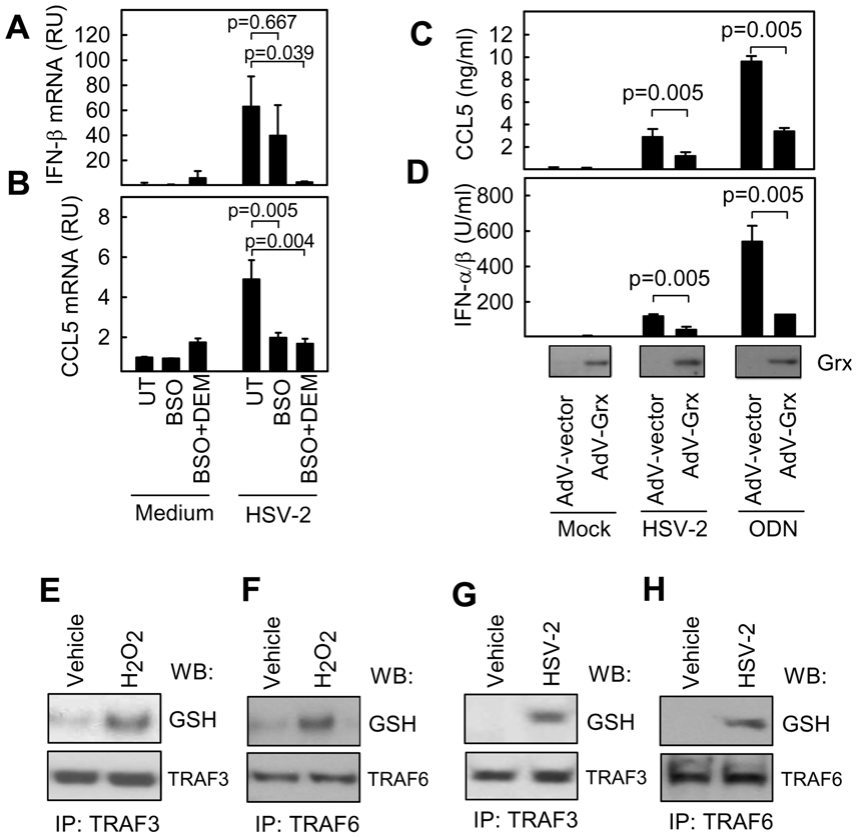
S-glutathionylation of TRAFs in response to exogenous and virus-induced ROS. (A, B) Peritoneal macrophages were treated overnight with buthionine sulfoximine (BSO, 200 µM). Diethylmaleate (DEM, 200 µM) was added 1 h prior to infection with HSV-2 (3×10^6^ pfu/ml, MOI 3). Total RNA was harvested 6 h p.i., and IFN-β and CCL5 mRNA was measured by real-time PCR. RNA levels were normalized against β-actin, and data shown as means relative units compared to untreated controls +/- st.dev. RU, relative units. (C, D) RAW264.7 cells were infected with AdV-empty vector or AdV-Grx for 2 days (MOI 10) before stimulation with HSV-2 (3×10^6^ pfu/ml, MOI 3) or ODN1826 (1 µM) as indicated. Supernatants were harvested 16 h post-stimulation and levels of IFN-α/β and CCL5 were measured. The data represent mean of triplicate cultures +/- st.dev. (E–H) RAW264.7 cells were treated with H_2_O_2_ (10 µM) or infected with HSV-2 (1×10^7^ pfu/ml, MOI 10) for 4 h. Total cell lysates were subjected to immunoprecipitation of TRAF3 and TRAF6, and the precipitates were blotted and probed with anti-glutathione antibodies.

**Figure 5:**
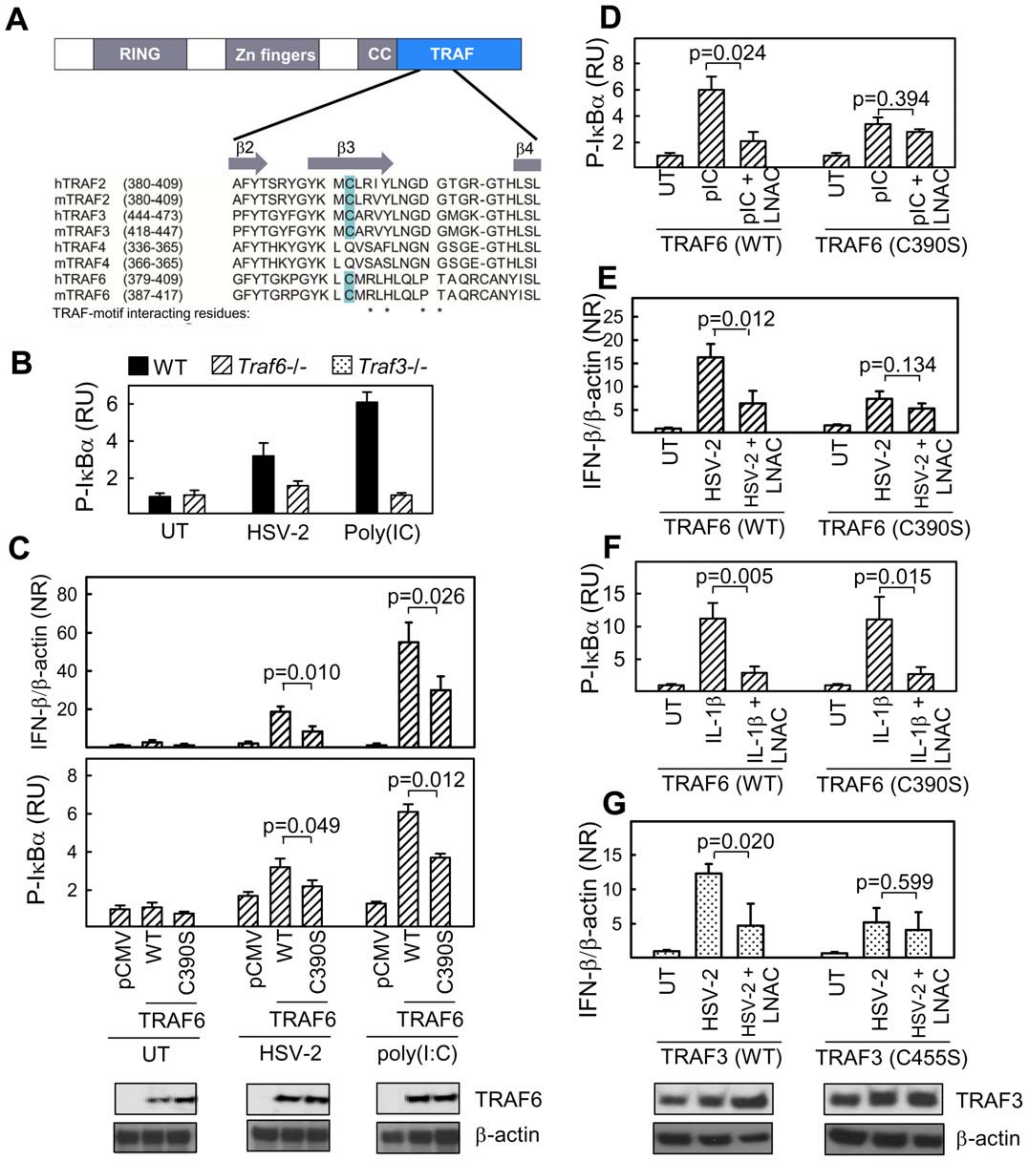
Identification of a conserved redox-sensitive surface-exposed cysteine in the TRAF C-terminal domain of TRAF2, 3, and 6. (A) Structure-based sequence alignment of a region of the TRAF domain of human and murine TRAF2, 3, 4, and 6. Secondary structures in the region and TRAF-motif interacting residues are indicated in the figure. (B) *Traf6-/-* MEFs were left untreated or treated with HSV-2 (3×10^6^ pfu/ml, MOI 3, 5 h) or poly(I∶C) (25 µg/ml, 2 h). Total cellular lysates we generated and phospho-IκBα was measured by Luminex. (C–F) Traf6-/- MEFs were transfected with empty vector (pCMV), WT TRAF6, or C390S TRAF6. The cells were treated with HSV-2 (3×10^6^ pfu/ml, MOI 3, 5 h), poly(I∶C) (25 µg/ml, 2 h), IL-1β (10 ng/ml), and LNAC (3.2 mM, 30 min prior to further treatment) as indicated. Total RNA and total cell lysates were isolated and analyzed for IFN-β mRNA and P-IκBα by RT-qPCR and Luminex, respectively. Data is shown as means of triplicates +/- st.dev. (G) *Traf3-/-* MEFs were transfected with empty vector (pRK5), WT TRAF3, or C455S TRAF3. The cells were treated with HSV-2 (3×10^6^ pfu/ml, MOI 3) and LNAC (3.2 mM, 30 min prior to further treatment) as indicated. Total RNA was isolated 5 h post-infection and analyzed for IFN-β mRNA by RT-qPCR. Data is shown as means of triplicates +/- st.dev. RU, relative units; NR, normalized ratio.

ROS can influence signal transduction through many mechanisms [16], [29], [30]. However, one of the most important mechanisms involves reversible S-glutathionylation of cysteine residues that alters protein function either by changing the protein active site or modifying protein-protein interactions [20]. To address whether S-glutathionylation could be involved in the positive stimulatory roles of ROS on innate immune signaling, we treated cells with the GSH-depleting agents buthionine sulfoximine and diethylmaleate prior to infection with HSV-2 [31]. Diminution of cellular GSH levels decreased the ability of HSV-2 to induce IFN-β and CCL5 mRNA expression (Figure 4A, B). To further address the potential role of S-glutathionylation in ROS-dependent positive stimulation of innate immune signaling, we transduced RAW264.7 cells with recombinant adenovirus (AdV) expressing glutaredoxin (Adv-Grx) 1, in order to decrease S-glutathionylation, or with an empty vector adenovirus (Adv-vector). Glutaredoxin overexpression decreases S-glutathionylation of proteins by catalyzing deglutathionylation freeing the protein thiol groups. Infection of the cells with HSV-2 or stimulation with the TLR9 ligand ODN1826 led to accumulation of CCL5 and IFN-α/β in the culture supernatants of the cells expressing AdV-vector and this response was significantly decreased in cells expressing Adv-Grx (Figure 4C, D). TRAF family proteins have been reported to be sensitive to redox-dependent regulation of signal transduction [16], [32], and we therefore examined whether ROS affected the glutathionylation of TRAF3 and TRAF6, which are critically involved in activation of pathways upstream of IRF-3 and NF-κB, respectively. Interestingly, H_2_O_2_ treatment, which elevated HSV-induced cytokine expression (Figure 1B–D), led to a clear increase in GSH moieties associated with both TRAF3 and TRAF6 (Figure 4E, F). Importantly, glutathionylation of TRAF 3 and 6 was also observed following HSV-2 infection (Figure 4G, H). Structure-based sequence alignment of TRAF family proteins revealed a cysteine residue in the β3 sheet in the TRAF C-terminal domain, which is conserved between TRAF2, 3, and 6, in both rodents and humans (Figure 5A). Examination of the structure of the TRAF6 C-terminal domain complexed with a peptide from RANK (PDB ID 1LB5) showed that this residue (Cys390) is surface-exposed and localized in close proximity to the peptide-binding pocket [33]. We docked GSH onto the structure of the TRAF6 C-terminal domain in the proximity of Cys390. The docking revealed that the structure was compatible with glutathionylation of Cys390, as the only clash was with Arg466 (data not shown). However, this clash could be relieved by changing the side chain conformation of Arg466, directing it further towards the RANK binding pocket. Thus, glutathionylation of Cys390 and contact between the GSH group and the binding partners of TRAF6 is in accordance with the current structural knowledge.

To determine the role of this potential glutathionylation site in TRAF6 mediated signaling, wild type TRAF6 or TRAF6 C390S mutant were transfected into *Traf6*-/- fibroblasts and P-IκBα monitored in response to treatment with HSV-2 or poly(I∶C). *Traf6*-/- fibroblasts displayed reduced P-IκBα compared to wild type fibroblasts in response to both HSV-2 infection and treatment with poly(I∶C) (Figure 5B). Reconstitution of *Traf6*-/- fibroblasts with wild type TRAF6 returned HSV-2 and poly(I∶C)-stimulated P-IκBα to levels consistent with wild type fibroblasts (Figure 5B, C). However, reconstitution of *Traf6*-/- fibroblasts with TRAF6 C390S was unable to completely rescue P-IκBα and IFN-β mRNA induction in response to HSV-2 and poly(I∶C) (Figure 5B, C). Furthermore, cells transfected with the C390S mutant were less sensitive to LNAC treatment than cells transfected with WT TRAF6 after poly(I∶C) treatment and HSV-2 infection, as measured by IκBα phosphorylation and IFN-β mRNA (Figure 5D, E). By contrast, the ability of IL-1β to activate the NF-κB pathway was not compromised in TRAF6 C390S-expressing cells, despite strong sensitivity towards LNAC treatment (Figure 5F), hence suggesting differences in the mode of action of ROS in different signaling pathways. Finally, introduction of a Cys-to-Ser mutation at position 455 in TRAF3, which aligned with Cys390 of TRAF6, led to reduced induction of IFN-β by HSV-2 after transfection into *traf3-/-* MEFs, and importantly abolishment of the sensitivity towards LNAC treatment, which was found in *traf3-/-* cells transfected with WT TRAF3 (Figure 5G). Collectively, these data indicate that HSV infection leads to production of ROS, which is essential for activation of innate antiviral immune responses, and this proceeds via a mechanism involving S-glutathionylation of TRAF family proteins.

## Discussion

The innate immune response constitutes the first component of the host defense machinery against pathogens. Exposure to an invading pathogen triggers recruitment and activation of phagocytic cells that initiate a respiratory burst, consisting in a robust production of ROS, in order to eliminate the invading pathogen. However, it is increasingly believed that moderate intracellular concentrations of ROS can act as a second messenger involved in the activation of innate immune signaling pathways against pathogens. In this study, we demonstrate that ROS are produced early during HSV infection and are essential for triggering an effective antiviral response against HSV *in vivo* and *in vitro*, involving type I IFNs, CXCL10 and CCL5 secretion.

There are several reports describing ROS production induced by viral infection, such as hepatitis C virus, influenza A, respiratory syncytial virus, and Sendai virus [34]–[37]. Lipopolysaccharide also stimulates ROS production via NOX and xanthine oxidase [30], [38]. Due to the high reactivity attributed to ROS, these radicals can quickly interact with the surrounding macromolecules. This has been seen during pulmonary H5N1 influenza A virus infection where ROS production generates oxidized phospholipids, which are TLR4 agonists playing a key role in lung injury during infection [36]. The pro-inflammatory response triggered by H5N1 infection can be reduced with LNAC treatment and as a result reduce lung injury [39]. Likewise, it has been previously reported that HSV infection-induced ROS are responsible for the formation of lipid peroxidation byproducts and neurotoxicity, characteristic of encephalitis [40], [41]. Thus, ROS are not only produced by macrophages and neutrophils to play a part in the killing of invading pathogens, but are a key component for the activation of PRR signaling.

In the case of HSV, several PRRs are involved in the recognition of the virus [14], including membrane-bound TLRs, and cytoplasmic RLRs and DNARs [3], [6], [7], [9], [12], [13], [42]. Here, we have examined the role of ROS in induction of innate immune responses by HSV as well as all the reported receptors recognising HSV. The data demonstrate that ROS are necessary for optimal signaling and gene expression during HSV infection or stimulation of PRRs. Most notably, the recently identified DNA receptor IFI16 senses HSV DNA in the cytoplasm and initiates a signaling cascade that is also redox-sensitive [7]. However, the sources of ROS might be different for the various receptors since they are located in different cellular compartments. It has previously been reported from separate groups that increased mitochondria-derived ROS production inhibits TLR4-mediated NF-κB activation [43], [44], whereas cytoplasmic NOX-derived ROS positively affects this activity [30]. Woo *et al*. have recently shed some light to this problem. They have shown that cells stimulated via immune receptors, such as T and B cell receptors, inactivate membrane-bound peroxiredoxin I (PrxI) and not cytoplasmic PrxI [45]. This allows transient localized accumulation of H_2_O_2_ around the membranes where signaling molecules such as protein tyrosine phosphatases are being recruited to the activated receptor [45]. Consequently, this intracellular oxidant gradient allows a regulated management of the signaling to the otherwise uncontrolled radical interactions, due to the nature of ROS.

The recognition of viruses is coordinated at the subcellular level by triggering signaling pathways leading to activation of transcription factors, and consequent expression of antiviral cytokines and chemokines. All these processes are regulated at the post-transcriptional level, with phosphorylation being the best described. However, other post-translational modifications have gained increasing importance, such as oxidation, ubiquitination, SUMOylation or glutathionylation. In this work we show that HSV oxidative-dependent activation of NF-κB and IRF-3 pathways plays a key role downstream of the PRRs recognizing HSV. NF-κB was the first transcription factor recognized to be redox-regulated [46]. ROS not only activates NF-κB through promotion of the degradation of IκBα, but also induces several post-translational modifications of the NF-κB subunit p65 that are necessary for the transcription of NF-κB-dependent pro-inflammatory genes [47]. TNF-α-induced IL-8 gene expression in monocytes is mainly controlled at the transcriptional level through NF-κB although redox-sensitive phosphorylation at Serine 276 of p65 is required [48]. For the IRF-3 pathway, different outcomes have been described in redox-dependent activation of IRF-3. It has been reported that NOX-derived ROS stimulate activation of the non-canonical IKK-like kinase, IKKε, and so IRF-3 activation, during infection with respiratory syncytial virus [34]. Recently, it has been shown that NOX2-derived ROS are critical for efficient RIG-I-mediated activation of IRF-3 and induction of IFN expression [37], whereas activation of this pathway through TLR4 is dependent of NOX4-derived ROS and ASK1 [26]. Prinarakis *et al*., on the other hand reported that IRF-3 is constitutively S-glutathionylated in HEK293 cells and is deglutathionylated by Grx upon Sendai virus infection, which is essential for the ability of IRF-3 to promote transcription [49]. In the primary murine peritoneal macrophages used in this study, constitutive glutathionylation of endogenous IRF-3 was not detected (data not shown), but our data do not exclude that ROS enhance signaling at the level of IKKε and MAVS. Collectively, the literature on ROS and activation of PRRs suggest that ROS interacts with signaling at multiple steps, which may implicate that the subcellular sources of ROS as well as the microenvironment in which a PPR operates (e.g. endosomes and mitochondrial surfaces) impacts on the role and mechanism of action of ROS in innate immunity. This adds a layer of complexity to the already intricate signaling of the innate immune response.

S-glutathionylation has also been suggested to be an important player in redox signaling [20], [31]. We found that depletion of cellular GSH levels or reversing of S-glutathionylation by overexpression of Grx strongly inhibited HSV-induced expression of IFNs and ISGs. Moreover, HSV-2 infection led to detectable glutathionylation of both TRAF3 and TRAF6, which was also seen in response to the potent ROS stimuli H_2_O_2_. Finally, a conserved surface-exposed cysteine residue is presented in the TRAF domain of TRAF2, 3, and 6, which is involved in redox-sensitive signaling pathways from PRRs and cytokine receptors [2], but not in TRAF4, which is generally believed not to be involved in these pathways [50]. We found that mutation of this cysteine in TRAF3 and 6 decreased the signaling and cytokine response to HSV-2 infection as well as the redox-sensitive nature of the response. Interestingly, a recent study demonstrated that human glutathione S-transferase P1-1 interacts with the TRAF domain of TRAF2 and specifically down-regulates signaling to MAPKs [32], which together with our present findings suggests that S-glutathionylation of TRAFs or associated protein represents a mechanism by which ROS re-direct and amplify signaling in the innate immune system. In contrast to this model, a recent report based on Grx-1 knock-down in 293HEK and Hela cells suggested that TRAF6 gets deglutathionylated in the RING finger motif upon IL-1/TLR stimulation, which was proposed to be essential for subsequent activation [51]. Therefore, with the available data, it is clear that we are still at an early stage in our understanding of the role of S-glutathionylation in immunological signal transduction.

In conclusion, we provide evidence that HSV infection quickly induces intracellular ROS which are necessary for proper activation of innate antiviral immune responses. The stimulatory functions of ROS appear to be mediated through S-glutathionylation, and we suggest TRAF family proteins to be important targets in positive ROS signaling. Recently, novel important roles for ROS in the innate immune system have been described, including inflammasome activation and programmed necrosis [28], [52]. Thus, it is becoming increasingly evident that ROS are key players in the host response to infection and inflammation, and that further understanding of the molecular details underlying the production and action of ROS may provide important knowledge on antiviral response mechanisms and pathogenesis of many human diseases.

## Materials and Methods

### Ethics statement

This study was carried out in accordance with the recommendations in the Guide for Care and Use of Laboratory animals, Institute of Laboratory Animal Resources, National Academy press (1996). All animal experiments were done in accordance with a protocol (permit number 2009/561-1613), which was approved by The Danish Committee for Animal Research (Ministry of Justice).

### Mice

C57BL/6, TLR9^-/-^, TLR2/9^-/-^ and ASK1^-/-^ mice [11], [53], [54] were bred at M&B Taconic (Laven, Denmark) and kept in the animal facility at The Faculty of Health Sciences, AU between the time of delivery (at 4 to 6 weeks of age) and the time of the experiments, and used for experiments between the age of 7 and 9 weeks old.

### Cells

Peritoneal cells were harvested by lavage of the peritoneal cavity with PBS containing 5% foetal calf serum (FCS) and 20 IU/ml heparin. Cells were washed, counted, and re-suspended in RPMI 1640-5% FCS for sub-culturing. For *in vitro* experiments, the cells were used at a concentration of 3.0×10^6^ cells per well in 96-well plates in 100 µl of RPMI 1640 with 5% FCS. BMMs were obtained as follows: femurs and tibia were surgically removed from C57BL/6 and MAVS^-/-^ mice, freed of muscles and tendons, and briefly suspended in 70% ethanol. Ends were cut, the marrow was flushed with 10% RPMI 1640, and cell suspension was filtered over a 70-µm cell strainer (BD Falcon) and centrifuged for 5 min at 1330 rpm. After 2 washes, cells were resuspended at 2×10^5^/ml in RPMI 1640 with 10% FCS and GM-CSF (10 ng/ml) and seeded in bacteriological petri dishes and incubated at 37°C with 5% CO_2_ and media changed after 3 and 5 days. On day 7, adherent cells were harvested from the dishes with medium containing 10 ng/ml GM-CSF. The cells were centrifuged, washed, and resuspended in RPMI 1640, 10% FCS, and GM-CSF (20 ng/ml), and examined by flow cytometry for expression of showing CD11b and CD11c (data not shown). For *in vitro* experiments, the cells were used at a concentration of 1.0×10^6^ cells per well in 96-well plates in 100 µl medium. To isolate primary pDCs cells from spleens from C57BL/6 and TLR9^-/-^ mice, organs were surgically removed and transferred to RPMI 1640 with 5% FCS. The spleens were then transferred to a 1 mg/ml suspension of collagenase D (Roche). The enzyme was injected into the organ, which was subsequently cut into small pieces, followed by incubation in collagenase D suspension for 30 min at 37°C. The suspension was filtered over a 70-µm pore size cell strainer, spun down, and suspended in RPMI 1640–5% FCS, and the cells were counted. After centrifugation, the cells were resuspended in PBS with 2 mM EDTA-0.5% BSA (MACS running buffer) in accordance with the manufacturer's instruction (Miltenyi Biotec). Anti-mPDCA-1 microbeads were added, and after incubation for 15 min at 4°C the suspension was spun down and suspended in running buffer. pDCs were then isolated in an autoMACS separator by positive selection. For *in vitro* experiments, the cells were used at a concentration of 1.0×10^6^ cells per well in 96-well plates in 100 µl RPMI 1640 with 5% FCS. RAW264.7 cells and MEFs (C57BL/6, traf3-/- (G. Cheng, LA, CA, USA) and traf6-/- (T. Mak, Toronto, Canada) were grown in DMEM supplemented with 5-10% FCS.

### Reagents

The oxidant-sensitive dye 5-(and-6)-chloromethyl-2′,7′-dichlorodihydrofluorescein diacetate (CM-H_2_DCFDA) was purchased from Invitrogen. Recombinant cytokines used for ELISAs were purchased from R&D Systems. The PRR agonists Poly(IC):LyoVec, Poly(I:C), Pam_3_Csk_4_, and ODN1826 were obtained from InvivoGen, IL-1β was from R&D Systems, and the HSV-1 dsDNA 60mer, described earlier [7], was from DNA Technology. Activation and inhibition of ROS production and function was achieved using H_2_O_2_ (Sigma-Aldrich), LNAC (Sigma Aldrich), PDTC (Sigma-Aldrich), and for GSH depletion was used buthionine sulfoximine (Fluka), diethylmaleate (Sigma-Aldrich). All small molecule inhibitors were used in concentrations that did not affect cell viability as determined by annexin V and propidium iodide staining. The pCMV hTRAF6 and pRK5 hTRAF3 expression plasmids were kindly provided by Andrew G. Bowie (Trinity College, Dublin). The TRAF6 C390S mutant was generated using the quick change kit (Stratagene) as described by the manufacture. The primers used were as follows. Forward: 5′-CCCGGGTACAAACTGTCCATGCGCTTGCACCTTCAGTTACCG-3′, Reverse: 5′-CGGTAACTGAAGGTGCAAGCGCATGGACAGTTTGTACCCGGG-3′. The TRAF3 C455S mutant was generated using a similar approach and the following primers; Forward: 5′-GGCTATAAGATGTCTGCCAGGGTCTACC-3′, Reverse: 5′-GGTAGACCCTGGCAGACATCTTATAGCC-3′. Oligonucleotides for HSV-1 60mer were synthesised by MWG Biotech; sequence is as follows: 5′-TAAGACACGATGCGATAAAATCTGTTTGTAAAATTTATTAAGGGTACAAATTGCCCTAGC-3′. Forward and reverse strands were annealed by heating for 5 min to 95°C and cooling to room temperature.

### Viruses and infections in vivo

The viruses used were HSV-1 (KOS strain), HSV-2 (MS strain), Sendai virus (Cantrell strain), AdV empty vector, and AdV-Grx [55]. Viruses were propagated and quantified as previously described [13], [55]. For infections *in vivo*, mice were injected intraperitoneally with 5×10^6^ pfu of HSV-2 as described previously [13]. At later time-points, serum, peritoneal cells, and livers were harvested for further analyses as described below. For modulation of ROS levels *in vivo*, mice were treated i.p. with LNAC (1.1 mmol/kg body weight) in saline 4 h prior to virus infection.

### Virus plaque assay

Samples of snap-frozen livers were weighed, thawed, and homogenized three times for 5 s in MEM supplemented with 5% FCS. After homogenization the organ suspensions were pelleted by centrifugation at 1,620 x *g* for 30 min, and the supernatants used for analysis. Plaque assays were performed on Vero cells as described previously [11].

### RNA-mediated interference

The siRNAs were chemically synthesised by Qiagen (p204-specific siRNA, 5′-CGGAGAGGAAUAAAUUCAUTT-3′; control siRNA, 5′-UUCUCCGAACGUGUCA CGUTT-3′). RAW264.7 cells were seeded in 12-well plates at a density of 1×10^5^ cells per well and were transfected with siRNA at a concentration of 12.5 pmol/ml with Lipofectamine 2000 (1 µl/ml). The cells were treated twice with siRNA on consecutive days and were grown for a further 48 h before stimulation. The degree of p204 knock-down was between 50 and 80%.

### Confocal microscopy

RAW 264.7 macrophages were plated at 1×10^5^ cells per well onto 10mm round coverslips. Cells were treated with inhibitors and infected with HSV-1 for 3 hr at 37°C, fixed with 4% formaldehyde (10 min, room temperature), permeabilised with methanol (90 sec at ÷20°C) and blocked with 5% normal goat serum (15 min, room temperature). Cells were incubated with polyclonal rabbit anti-IRF-3 (Santa Cruz Biotechnology, CA, USA) at room temperature for 1 hr, and counterstained with Alexa Fluor 568 conjugated anti-rabbit antibodies (Molecular Probes) for an additional 1 hr at room temperature. Finally, cells were stained with DAPI and coverslips mounted in Pro-Long Gold (Molecular Probes). Images were collected using Zeiss LSM710 confocal microscope 63 x oil objective. For quantification of cells positive for nuclear IRF-3, regions of interest were identified based on DAPI staining, and levels of nuclear IRF-3 was determined. An arbitrary threshold was set, and percentage of positive cells in each group was calculated. Between 95 and 150 cells were counted for each condition and scored for IRF-3 localization.

### Live-microscopy

RAW 264.7 macrophages were seeded at 5×10^4^ cells per well on a 8-wells chambered coverglass (NUNC). Cells were either pre-treated with 6,4 mM LNAC for 30 min or with vehicle and subsequently infected with HSV-2 for 1, 2, or 3 hours. Media was then changed to a Phenol red-free DMEM and transferred to 37°C warmed heated-chamber of the confocal microscope. Cells were then incubated with 5 µM CM-H_2_DCFDA and imaged immediately after as described by manufacturer using Zeiss LSM710 confocal microscope 40x or 20x objectives. Z-stack images were taken (4–5 slices of 1 µm size) at every 30 seconds for up to 1,5 min. For quantification of ROS production, background fluorescence was eliminated and total fluorescence was measured in each frame using Image J software. The number cells per frame was calculated and a minimum of 100 cells were counted per condition. Amount of ROS was represented as fluorescence per cell.

### ELISA and Luminex

Cytokine measurements were carried out using ELISAs based on matched antibody pairs from R&D Systems as described [13]. Levels of phosphorylated IκBα was determined using Luminex technology and kits from Bio-Rad, and following the instructions from the manufacturer.

### Type I IFN bioassay

The bioactivity of type I IFN in culture supernatants and serum was determined using a L929-cell based bioassay as described previously [11]. High levels of IFN-γ or IFN-λ did not interfere with the assay.

### Quantitative RT-PCR

Total RNA from human and murine macrophages was purified using the High Pure RNA Isolation kit from Roche. For cDNA generation, RNA was subjected to reverse transcription with oligo(dT) as primer and Expand reverse transcriptase (Roche). For data from Figure 3D, RNA was subjected to one-step qPCR (Stratagene). For measurement of cytokines, the cDNA was PCR-amplified using the following primers: IFN-β, forward: 5′-CACTGGGTGGAATGAGACTAT-3′,reverse: 5′-GACATCTCCCACGTCAATC-3′, CCL5, forward: 5′-ACTCCCTGCTGCTTTGCCTAC-3′, reverse: 5′-GCGGTTCCTTCGAGTGACA-3′, β-actin, forward: 5′-TAGCACCATGAAGATCAAGAT-3′,reverse: 5′-CCGATCCACACAGAGTACTT-3′. Products were measured using SYBR Green I (Qiagen) and normalized against β-actin. For processing of data, we selected the cycle threshold value of samples and normalized to β-actin following standard procedures. Data were presented as relative units compared to the average of untreated controls

### Immunoprecipitation and Western blotting

Cells were washed twice in phosphate-buffered saline, and lysed in 850 µl of lysis buffer (50 mM HEPES, pH 7.5, 150 mM NaCl, 2 mM EDTA, 10% (v/v) glycerol, 1% (v/v) Nonidet P-40, 0.5 mM DTT, Complete protease inhibitor (Roche), 0.5 mM Na_3_VO_4_, 20 mM N-ethylmaleimide, and 50 mM α-Iodoacetoamide). For immunoprecipitation, rabbit polyclonal anti-TRAF3 and rabbit polyclonal anti-TRAF6 (Santa Cruz Biotechnology, CA, USA) were precoupled to protein A/G-Agarose beads (Santa Cruz Biotechnology, CA, USA) overnight at 4°C. The beads were washed twice in lysis buffer and incubated with 1.5 mg of cell lysate per sample overnight at 4°C. The immune complexes were washed 3 times in lysis buffer, boiled, and analyzed by standard SDS-PAGE and Western blotting techniques using rabbit polyclonal anti-TRAF3/6 (as above), mouse monoclonal anti-glutathione (ViroGen, Watertown, VA, USA), and rabbit polyclonal anti-glutaredoxin-1 antibodies (Santa Cruz Biotechnology, CA, USA) for blotting.

### Molecular modeling

One molecule of glutathione was docked onto C390 in the published structure of TRAF6 (1LB5 and 1LB6) using the program Coot. This was possible with only minor changes of side chain conformation. The docking checked for steric clashes using the built-in routines in Coot. Coordinates for glutathione was taken from the Hicup server.

### Statistical analysis

The data are presented as means ± SD. The statistical significance was estimated with the Student t-test or Wilcoxon rank sum test (*p* values of <0.05 were considered to be statistically significant).

### Reproducibility of data

The results shown in this work are derived from data that are representative for the results obtained. For each series of experiments, two to six independent repetitions were performed.
